# DNAdesign: feature-aware *in silico* design of synthetic DNA through mutation

**DOI:** 10.1093/bioinformatics/btaf052

**Published:** 2025-01-31

**Authors:** Yingfei Wang, Jinsen Li, Tsu-Pei Chiu, Nicolas Gompel, Remo Rohs

**Affiliations:** Department of Quantitative and Computational Biology, University of Southern California, Los Angeles, CA 90089, United States; Department of Quantitative and Computational Biology, University of Southern California, Los Angeles, CA 90089, United States; Department of Quantitative and Computational Biology, University of Southern California, Los Angeles, CA 90089, United States; Department of Evolutionary Biology and Ecology, Bonn Institute for Organismic Biology, University of Bonn, Bonn 53115, Germany; Department of Quantitative and Computational Biology, University of Southern California, Los Angeles, CA 90089, United States; Department of Chemistry, University of Southern California, Los Angeles, CA 90089, United States; Department of Physics & Astronomy, University of Southern California, Los Angeles, CA 90089, United States; Thomas Lord Department of Computer Science, University of Southern California, Los Angeles, CA 90089, United States; Division of Medical Oncology, Department of Medicine, University of Southern California, Los Angeles, CA 90033, United States

## Abstract

**Motivation:**

DNA sequence and shape readout represent different modes of protein–DNA recognition. Current tools lack the functionality to simultaneously consider alterations in different readout modes caused by sequence mutations. DNAdesign is a web-based tool to compare and design mutations based on both DNA sequence and shape characteristics. Users input a wild-type sequence, select sites to introduce mutations and choose a set of DNA shape parameters for mutation design.

**Results:**

DNAdesign utilizes Deep DNAshape to provide ultra-fast predictions of DNA shape based on extended *k*-mers and offers multiple encoding methods for nucleotide sequences, including the physicochemical encoding of DNA through their functional groups in the major and minor groove. DNAdesign provides all mutation candidates along the sequence and shape dimensions, with interactive visualization comparing each candidate with the wild-type DNA molecule. DNAdesign provides an approach to studying gene regulation and applications in synthetic biology, such as the design of synthetic enhancers and transcription factor binding sites.

**Availability and implementation:**

The DNAdesign webserver and documentation are freely accessible at https://dnadesign.usc.edu.

## 1 Introduction

Protein–DNA interaction plays a pivotal role in many biological processes. Transcription factors (TFs) are DNA binding proteins that are involved in gene regulation. DNA sequence and shape contribute to a TF’s specific recognition of its DNA binding site (Garvie and Wolberger 2001, Mitra *et al.* 2024). Biophysically, proteins use two different readout modes: base readout and shape readout (Rohs *et al.* 2010). DNA base readout refers to hydrogen bond formation or hydrophobic interactions between amino acid side chains and the chemical groups of DNA bases whereas DNA shape readout is characterized by the recognition of 3D DNA structure. An example is the narrowing of the minor groove, which results in enhanced negative electrostatic potential that attracts positively charged amino acids such as arginine, lysine, or protonated histidine (Rohs *et al.* 2009, Jiang *et al.* 2024). Alteration of protein–DNA interaction through DNA shape changes is a biophysical mechanism through which nucleotide mutations lead to functional or phenotypic changes. For example, DNA shape-changing single nucleotide polymorphisms (SNPs) were found to be under selection pressure (Wang *et al.* 2018).

Despite our biophysical understanding of base and shape readout mechanisms, only a limited number of studies considered DNA sequence and shape when designing DNA oligos to study the functional significance of mutations in enhancers (Le Poul *et al.* 2020). To facilitate the integration of the two readout modes in experiments such as optimizing TF binding sequence and designing synthetic enhancers, we introduce DNAdesign, a web-based tool that enables the interactive design and comparison of candidate mutants. DNAdesign enables researchers to design oligos based on the desired sequence and shape changes with versatile customization options. DNAdesign enables users to choose from 14 DNA shape features predicted by Deep DNAshape, which is a deep learning approach that considers extended *k*-mers (Li *et al.* 2024).

DNAdesign also offers multiple approaches to encode nucleotide sequences when calculating base readout distances, including one-hot encoding and physicochemical encoding (Chiu *et al.* 2023). The physicochemical encoding represents DNA by the base pair-specific arrangement of functional groups (hydrogen bond acceptor and donor, nonpolar hydrogen, and methyl group) in the major and minor groove (Supplemental Fig. S1). DNAdesign offers a comprehensive overview of all candidates, presenting their shape and base-pair distances from the input sequence, with interactive features for candidate selection and visualization. DNAdesign aims to provide biologists with easily accessible structural and mechanistic insights and assists studies of enhancer function, synthetic biology, and gene regulation.

## 2 Implementation

DNA shape refers to a set of features that describe the 3D structural properties of the double helix. DNAdesign incorporates six inter-base pair, six intra-base pair, and two minor groove features, minor groove width (MGW) and electrostatic potential (EP) (see Supplementary Information). DNAdesign predicts DNA shape by utilizing the Deep DNAshape webserver (Li and Rohs 2024). Specifically, DNAdesign takes any input wild-type sequence of length *n* and utilizes the Deep DNAshape model to predict DNA shape features. Each inter-base pair parameter corresponds to a vector of length *n* −1, and each intra-base pair parameter and groove parameter is a vector of length *n*. The default DNA shape distance metric is the Euclidean distance. As an advanced setting, DNAdesign also offers Pearson's correlation as the alternative shape distance metric. This can be useful when it is crucial to preserve the pattern of a shape parameter along a sequence instead of the absolute values. A detailed definition is discussed in Supplementary Information.

**Figure 1.**
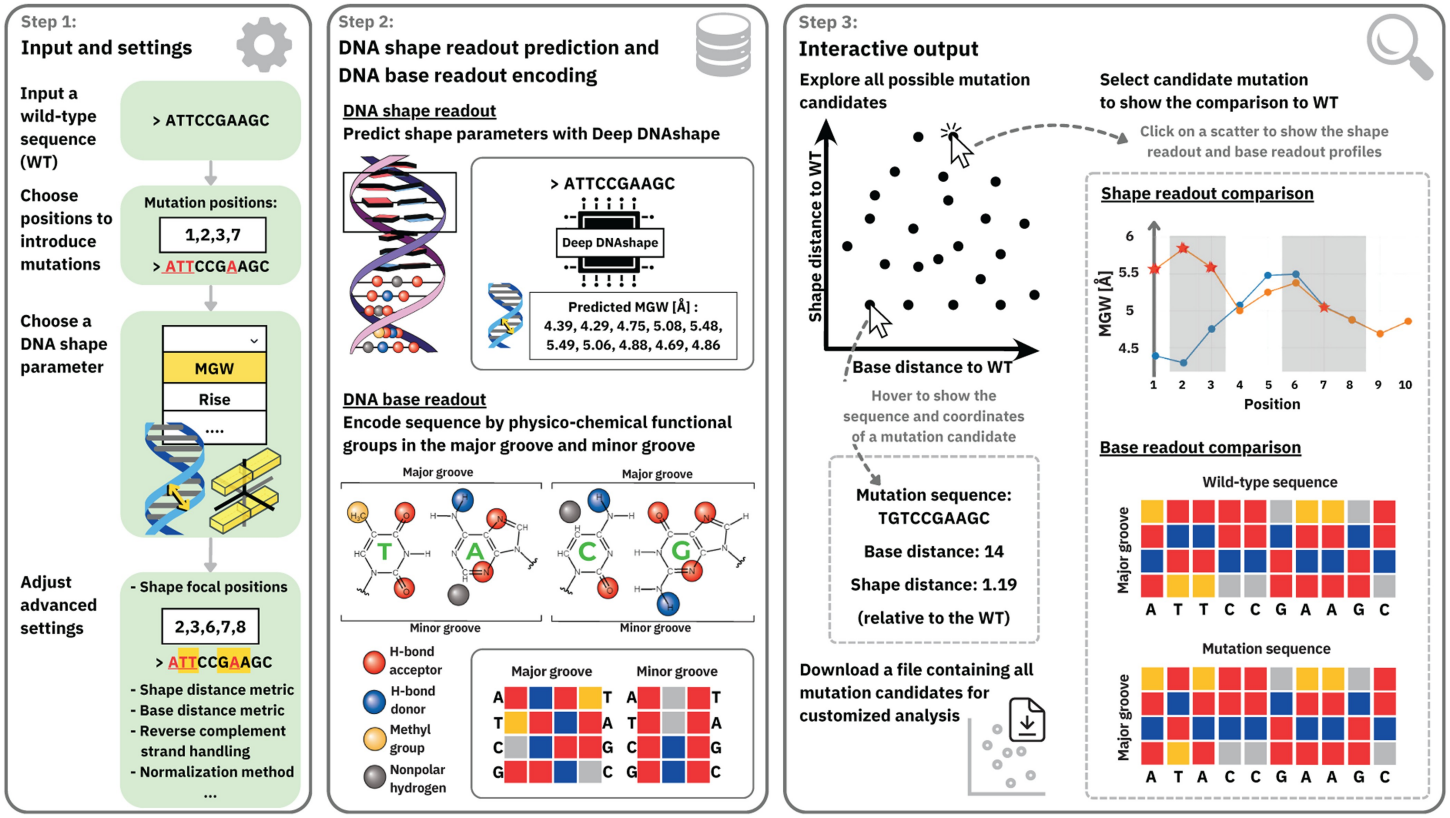
Illustration of the DNAdesign method and flowchart.

To quantify base readout alterations caused by sequence mutations, the default distance metric of DNAdesign is based on physicochemical encoding (Chiu *et al.* 2023). Specifically, each base pair is encoded by four functional groups on its major groove edge and three functional groups on its minor groove edge (Fig. 1; Supplementary Fig. S1). This approach is implemented through encoding of each functional group by a one-hot vector with length of four. Each base pair is represented by concatenating the functional group vectors into a major groove vector of length 16 and a minor groove vector of length 12. The base readout distance between an input sequence and a given mutation candidate is obtained by summing up the position-wise difference between the two base-pair feature matrices.

DNAdesign also offers alternative encoding methods and distance metrics for base-pair distance calculation including the one-hot encoding and the Levenshtein distance (see Supplementary Information). While the physicochemical encoding is equivalent to sequence one-hot encoding, the explicit description of functional groups allows for chemical modifications and base-pairing variations that deviate from the Watson-Crick geometry in a future extension of the method.

DNAdesign allows users to specify focal points for shape readout distance calculations. By default, readout features along the entire sequence length are used. However, since a point mutation will affect shape parameters in consecutive neighboring base pairs, selecting focal points for distance calculation enables users to study shape profile changes at specific nucleotides. This feature is particularly useful for designing DNA sequences to achieve desired shape patterns in specific areas of interest, such as the spacer region between cooperative TF binding sites or flanking sequences adjacent to a TF core binding motif.

The DNAdesign webserver is implemented with the Python Flask library as the backend. User input of DNA sequences, customization of options, and interaction with the graphs are handled by JavaScript and through the Plotly.js integration. Resulting figures can be downloaded from the webserver. For users who intend to save raw data to analyze offline, a downloadable file button can be found on the top right corner of the graphs. The webserver is designed to be interactive and easy to use. A detailed user guide and application examples can be found on the manual page.

## 3 Application

DNAdesign is a versatile tool for assisting experimental design for various research applications involving DNA sequence or shape. To illustrate its practical application, we demonstrate two case studies (Supplementary Figs S3 and S4).

In the first case study, we designed DNA sequences with increased binding affinities of the nucleoid protein Fis. Prior studies revealed that the DNA sequence affects Fis–DNA binding, particularly through sequence-dependent MGW (Hancock *et al.* 2013, Chiu *et al.* 2017). We input a DNA sequence with lower binding affinity to DNAdesign and identified three mutation candidates that optimize MGW. We found that all three candidates harbor the ideal mutation from C/G to A/T rich in the center of the binding site, including one candidate with experimentally verified higher binding affinity (Supplementary Fig. S3).

We also demonstrate that DNAdesign can be used to design shape-perturbing DNA oligos to test the DNA shape preference of a TF. A recent study investigated DNA binding of the Apicomplexan Apetala 2 (ApiAP2) TF family in the human malaria parasite (Bonnell *et al.* 2024). To test if changes in DNA shape readout will alter DNA binding affinity of a GAGCAC-binding ApiAP2 TF, the authors selected DNA oligos to maximize shape readout changes while controlling the number of point mutations. We showed that DNAdesign is perfectly suited to assist researchers with such design needs (Supplementary Fig. S4).

DNAdesign can also be applied to molecular genetics studies. For example, to investigate the regulatory logic of an enhancer, another recent study compared reporter gene expression patterns in transgenic *Drosophila* by substituting 7-base-pair A-tracts along a 196-base-pair enhancer region (Le Poul *et al.* 2020). DNAdesign can be used to systematically introduce DNA-shape perturbing mutations along the functional region or to target specific TF binding sites.

## 4 Conclusion

DNAdesign is a web-based application that enables the design of mutant DNA sequences with structural considerations. The webserver enables ultra-fast calculation of DNA shape profiles for up to 4^7^ mutation candidates at once. It allows users to customize distance metrics, select from 14 DNA shape features for analysis, and visualize and compare all mutation candidates in a single, easy-to-understand panel. DNAdesign incorporates an interactive display of shape and base readout profiles of the selected mutation candidate, providing an intuitive visualization. DNAdesign is currently limited to predicting local structural properties of unbound DNA, and it does not support mutations in more than seven nucleotide positions due to memory constraints.

With rapid advances in high-throughput structure predictions, our understanding of mutations is no longer limited to DNA sequence alone. An increasing number of studies demonstrate the functional significance of DNA structural and mechanical properties in biological processes (Basu *et al.* 2021a,b, Yao *et al.* 2024). Although tools such as AlphaFold 3 and RoseTTAFoldNA can predict 3D structures of protein–DNA complexes, their throughput and speed are limited (Abramson *et al.* 2024, Baek *et al.* 2024). Moreover, parsing these generated structures to obtain geometric descriptors of DNA is not straightforward. DNAdesign is designed to address this unmet need as an easy-to-use, complementary tool for researchers who want to consider both the sequence and structural aspects caused by DNA mutations, in a quick and low-barrier way. We envision that DNAdesign will further bridge the genomics and structural biology communities, enabling scientists to advance the exploration of the complex interplay between DNA sequence, structure, and function.

## Supplementary Material

btaf052_Supplementary_Data

## Data Availability

The source code for the DNAdesign webserver is available at https://github.com/wangyingfei/DNAdesign and for its release at https://zenodo.org/records/14036420.

## References

[btaf052-B1] Abramson J , Adler J, Dunger J et al Accurate structure prediction of biomolecular interactions with AlphaFold 3. Nature 2024;630:493–500. 38718835 10.1038/s41586-024-07487-w PMC11168924

[btaf052-B2] Baek M , McHugh R, Anishchenko I et al Accurate prediction of protein–nucleic acid complexes using RoseTTAFoldNA. Nat Methods 2024;21:117–21. 37996753 10.1038/s41592-023-02086-5 PMC10776382

[btaf052-B3] Basu A , Bobrovnikov DG, Ha T. DNA mechanics and its biological impact. J Mol Biol 2021a;433:166861. 33539885 10.1016/j.jmb.2021.166861

[btaf052-B4] Basu A , Bobrovnikov DG, Qureshi Z et al Measuring DNA mechanics on the genome scale. Nature 2021b;589:462–7. 33328628 10.1038/s41586-020-03052-3 PMC7855230

[btaf052-B5] Bonnell VA , Zhang Y, Brown AS et al DNA sequence and chromatin differentiate sequence-specific transcription factor binding in the human malaria parasite *Plasmodium falciparum*. Nucleic Acids Res 2024;52:10161–79. 38966997 10.1093/nar/gkae585 PMC11417369

[btaf052-B6] Chiu TP , Rao S, Mann RS et al Genome-wide prediction of minor-groove electrostatic potential enables biophysical modeling of protein–DNA binding. Nucleic Acids Res 2017;45:12565–76. 29040720 10.1093/nar/gkx915 PMC5716191

[btaf052-B7] Chiu TP , Rao S, Rohs R. Physicochemical models of protein–DNA binding with standard and modified base pairs. Proc Natl Acad Sci USA 2023;120:e2205796120. 36656856 10.1073/pnas.2205796120 PMC9942898

[btaf052-B8] Garvie CW , Wolberger C. Recognition of specific DNA sequences. Mol Cell 2001;8:937–46. 11741530 10.1016/s1097-2765(01)00392-6

[btaf052-B9] Hancock SP , Ghane T, Cascio D et al Control of DNA minor groove width and Fis protein binding by the purine 2-amino group. Nucleic Acids Res 2013;41:6750–60. 23661683 10.1093/nar/gkt357 PMC3711457

[btaf052-B10] Jiang Y , Chiu TP, Mitra R et al Probing the role of the protonation state of a minor groove-linker histidine in Exd-Hox–DNA binding. Biophys J 2024;123:248–59. 38130056 10.1016/j.bpj.2023.12.013 PMC10808038

[btaf052-B11] Le Poul Y , Xin Y, Ling L et al Regulatory encoding of quantitative variation in spatial activity of a *Drosophila* enhancer. Sci Adv 2020;6:eabe2955. 33268361 10.1126/sciadv.abe2955 PMC7821883

[btaf052-B12] Li J , Chiu TP, Rohs R. Predicting DNA structure using a deep learning method. Nat Commun 2024;15:1243. 38336958 10.1038/s41467-024-45191-5 PMC10858265

[btaf052-B13] Li J , Rohs R. Deep DNAshape webserver: prediction and real-time visualization of DNA shape considering extended *k*-mers. Nucleic Acids Res 2024;52:W7–12. 38801070 10.1093/nar/gkae433 PMC11223853

[btaf052-B14] Mitra R , Li J, Sagendorf JM et al Geometric deep learning of protein– DNA binding specificity. Nat Methods 2024;21:1674–83. 39103447 10.1038/s41592-024-02372-w PMC11399107

[btaf052-B15] Rohs R , Jin X, West SM et al Origins of specificity in protein–DNA recognition. Annu Rev Biochem 2010;79:233–69. 20334529 10.1146/annurev-biochem-060408-091030 PMC3285485

[btaf052-B16] Rohs R , West SM, Sosinsky A et al The role of DNA shape in protein–DNA recognition. Nature 2009;461:1248–53. 19865164 10.1038/nature08473 PMC2793086

[btaf052-B17] Wang X , Zhou T, Wunderlich Z et al Analysis of genetic variation indicates DNA shape involvement in purifying selection. Mol Biol Evol 2018;35:1958–67. 29850830 10.1093/molbev/msy099 PMC6063282

[btaf052-B18] Yao YM , Miodownik I, O'Hagan MP et al Deciphering the dynamic code: DNA recognition by transcription factors in the ever-changing genome. Transcription 2024;5:1–25. 10.1080/21541264.2024.2379161 PMC11810102 39033307

