# Supplementary material for: DNAdesign: feature-aware in silico design of synthetic DNA through mutation
Source: Bioinformatics. 2025 Jan 31;41(2):btaf052. doi: 10.1093/bioinformatics/btaf052 (PMC11825384; doi:10.1093/bioinformatics/btaf052)
Supplement: btaf052_Supplementary_Data [file btaf052_supplementary_data.pdf]

SUPPLEMENTARY DATA

DNAdesign: feature-aware *in silico* design of synthetic DNA through mutation

Yingfei Wang[1], Jinsen Li[1], Tsu-Pei Chiu[1], Nicolas Gompel[2], and Remo Rohs[1,3,4,5,6,*]

[1]Department of Quantitative and Computational Biology, University of Southern California, Los Angeles, CA 90089, USA

[2]Bonn Institute for Organismic Biology, University of Bonn, 53115 Bonn, Germany

[3]Department of Chemistry, University of Southern California, Los Angeles, CA 90089, USA

[4]Department of Physics and Astronomy, University of Southern California, Los Angeles, CA 90089, USA

[5]Thomas Lord Department of Computer Science, University of Southern California, Los Angeles, CA 90089, USA

[6]Division of Medical Oncology, Department of Medicine, University of Southern California, Los Angeles, CA 90033, USA

*To whom correspondence should be addressed: rohs@usc.edu

Supplementary Information

S1. DNA shape and base readout

In this work, DNA shape refers to a set of 14 metrics that describe the structural or biophysical measurements along the DNA double helix (Li *et al.*, 2017). The 14 DNA shape parameters include six intra-base pair parameters, six inter-base pair parameters, and two minor groove parameters. Intra-base pair parameters quantify the relative positions of the two bases in a base pair: shear, stretch, stagger, buckle, propeller twist (ProT), and opening. Inter-base pair parameters quantify the relative positions of two neighboring base pairs: shift, slide, rise, tilt, roll, and helical twist (HelT). The two minor groove parameters are minor groove width (MGW) and electrostatic potential (EP). MGW is the minimum distance between the phosphodiester backbones of two DNA strands measured at a specific base pair. A detailed definition is discussed in (Zhou *et al.*, 2013). EP is the predicted electrostatic potential at the center of the minor groove in the approximate plane a base pair. A detailed definition is discussed in (Chiu *et al.*, 2017).

For base readout, each base pair is represented by the functional groups at the minor and major groove edges of the bases that make direct contact with protein side chains. The functional groups include hydrogen bond donor, hydrogen bond acceptor, non-polar hydrogen, and methyl group (Chiu *et al.*, 2023).

S2. Advanced settings

DNAdesign provides a few advanced setting options: shape focal points, shape distance metric, shape distance normalization, base distance metric, base distance normalization, and reverse complement strand handling.

S2.1 Shape focal points

Users can choose specific positions as the shape focal points. DNAdesign allows both single-nucleotide position input and selection of a range of nucleotide positions. By default, DNAdesign uses shape parameters across the entire sequence to calculate the shape distance between wild-type and a specific mutation candidate sequence, for example, when using Euclidean distance:

$$d_{shape\_Mut} = \sqrt{\left(S_{1_{WT}} - S_{1_{Mut}}\right)^2 + \left(S_{2_{WT}} - S_{2_{Mut}}\right)^2 + \cdots \left(S_{n_{WT}} - S_{n_{Mut}}\right)^2}$$

where

$[S_{1_{WT}}, S_{2_{WT}}, \dots, S_{n_{WT}}]$ denotes the shape parameter of the wild-type sequence of length *n*, and $[S_{1_{Mut}}, S_{2_{Mut}}, \dots, S_{n_{Mut}}]$ denotes the shape parameter of a mutation candidate which is also of length *n*.

When the user inputs shape focal positions, the Euclidean distance is calculated as:

$$d_{shape\_Mut} = \sqrt{\left(S_{p1_{WT}} - S_{p1_{Mut}}\right)^2 + \left(S_{p2_{WT}} - S_{p2_{Mut}}\right)^2 + \cdots \left(S_{pm_{WT}} - S_{pm_{Mut}}\right)^2}$$

where

$\left[S_{p1_{WT}}, S_{p2_{WT}}, \dots, S_{pm_{WT}}\right]$ denotes the shape parameters that correspond to the $p1^{st}$, $p2^{nd}$, … ,pm^{th} position of the input wild type sequence, $\left[S_{p1_{Mut}}, S_{p2_{Mut}}, \dots, S_{pm_{Mut}}\right]$ denotes the shape parameters of a mutation candidate, and *p1,p2 … pm* correspond to user input shape focal positions. For example, if the input wildtype sequence is ATTCCGAT, the shape focal points are 2, 5, and 7, then:

$$d_{shape\_Mut} = \sqrt{\left(S_{2_{WT}} - S_{2_{Mut}}\right)^2 + \left(S_{5_{WT}} - S_{5_{Mut}}\right)^2 + \cdots \left(S_{7_{WT}} - S_{7_{Mut}}\right)^2}$$

where $[S_{2_{WT}}, S_{5_{WT}}, S_{7_{WT}}]$ denotes the shape parameters that correspond to the 2nd, 5th, and 7th positions of the wild-type sequence, and $[S_{2_{Mut}}, S_{5_{Mut}}, S_{7_{Mut}}]$ denotes the shape parameter of the 2nd, 5th, and 7th positions of a mutation candidate.

S2.2 Shape readout distance metrics

For shape distance metric, DNAdesign uses Euclidean distance as default and allows users to choose Pearson's correlation coefficient as alternate shape distance metric. Specifically:

$$d_{shape\_Mut\_pearson} = \frac{\sum_1^n \left(S_{i_{WT}} - \overline{S_{i_{WT}}}\right)\left(S_{i_{Mut}} - \overline{S_{i_{Mut}}}\right)}{\sqrt{\sum_1^n \left(S_{i_{WT}} - \overline{S_{i_{WT}}}\right)^2 \sum_1^n \left(S_{i_{Mut}} - \overline{S_{i_{Mut}}}\right)^2}}$$

where

$S_{i_{WT}}$ denotes the shape parameter corresponding to the ith position of the wild-type sequence, $\overline{S_{i_{WT}}} = \frac{S_{1_{WT}} + S_{2_{WT}} + \cdots + S_{n_{WT}}}{n}$. The same notation method is used for the mutation sequences. When shape focal points are specified, DNAdesign will only use the selected positions to calculate Pearson's correlation coefficient.

DNA shape parameters vary in scale, DNAdesign provides an option to display normalized shape distance in the scatter plot that compares all mutation candidates:

$$d_{shape\_Mut\_normalized} = \frac{d_{shape\_Mut} - \min(d_{shape\_Mut_j})}{\max(d_{shape\_Mut_j}) - \min(d_{shape\_Mut_j})}$$

Where $\min(d_{shape\_Mut_j})$ and $\max(d_{shape\_Mut_j})$ are the smallest and largest shape distances to the input wild type among all possible mutation candidates.

S2.3 Base readout representation and base readout distance calculation

For base readout representation, DNAdesign represents sequences numerically with the physicochemical encoding method by default, with four functional group positions in the major groove and three functional group positions in the minor groove (Chiu *et al.*, 2023):

Adenine (A):

Major groove (M): [0,0,0,1, 0,0,1,0, 0,0,0,1, 0,1,0,0],

Minor groove (m): [0,0,0,1, 1,0,0,0, 0,0,0,1],

Cytosine (C):

Major groove (M): [1,0,0,0, 0,0,1,0, 0,0,0,1, 0,0,0,1],

Minor groove (m): [0,0,0,1, 0,0,1,0, 0,0,0,1],

Guanine (G):

Major groove (M): [0,0,0,1, 0,0,0,1, 0,0,1,0, 1,0,0,0],

Minor groove (m): [0,0,0,1, 0,0,1,0, 0,0,0,1],

Thymine (T):

Major groove (M): [0,1,0,0, 0,0,0,1, 0,0,1,0, 0,0,0,1],

Minor groove (m): [0,0,0,1, 1,0,0,0, 0,0,0,1],

At any given position, the distance between two distinct base pairs is calculated as

$$d_{B_1,B_2} = \sum_{M=1}^{16} |B_{1_M} - B_{2_M}| + \sum_{m=1}^{12} |B_{1_m} - B_{2_m}|$$

where B_1 and B_2 are nucleotides among A, C, G, T, and $B_{1_M}, B_{1_m}, B_{2_M}, B_{2_m}$ are either 0 or 1, corresponding to the encoding shown above. M denotes the major groove encoding, and m denotes the minor groove encoding. For example,

$$d_{A,C} = |0-1| + |0-0| + |0-0| + |1-0| + \cdots + |1-1| = 6$$

Base distance between the wildtype sequence of length *n* and a mutation candidate is

$$d_{base\_Mut} = \sum_{i=1}^{n}(d_{WT_i,Mut_i})$$

where WT_i, Mut_i denotes the base corresponding to the ith position of the wild-type and mutation candidate sequence, respectively.

In addition to physicochemical encoding, DNAdesign also provides the user with the option to represent DNA with the one-hot encoding method:

A: [1,0,0,0], C: [0,1,0,0], G: [0,0,1,0], T: [0,0,0,1]

The base distance is calculated in the same way as described above, with $d_{B_1,B_2} = 2$ for all B_1 and B_2 when $B_1 \neq B_2$.

For base distance calculation, DNAdesign also provides users with the option to use the edit distance, or Levenshtein distance, which is the minimum number of insertions, deletions, or substitutions required to modify one sequence to another. DNAdesign calculates the Levenshtein distance using the Python C extension module *python-levenshtein*, publicly available through pip (Bachmann *et al.*, 2021).

Regardless of the base-pair encoding method and base-pair distance metric choice, users can choose to display the normalized base-pair distance on the global comparison graph. The normalization method is the same as in shape distance normalization.

S2.4 Additional settings

DNAdesign provides options to treat reverse complement sequence as either the same with or different from the input strand. This can be particularly useful when designing short DNA sequences for low-throughput experiments that utilize double-stranded DNA. We do not recommend using this option when designing mutations of a subsequence within a longer region. For example, when the goal is to design a series of mutation candidates that span a long enhancer sequence and use DNAdesign to devise mutations for each 10-base-pair sliding window.

S3. Application case studies

S3.1 Design a high-affinity Fis binding site

Fis is an abundant nucleoid protein that primarily interacts with DNA through backbone contact and identifies target sites by sensing DNA shape conformation. Research indicates that the DNA sequence affects Fis–DNA binding, particularly through the sequence-dependent MGW in the center and in flanking regions of the binding sites. Specifically, an A/T-rich center (nucleotide positions -2 to +2) is crucial due to its compressed minor groove, which shortens the distance between neighboring major groove regions and facilitates contacts between DNA and the recognition helices of Fis (Hancock *et al.*, 2013, 2016; Chiu *et al.*, 2017).

Based on the shape-sensing characteristics of Fis, one can use DNAdesign to devise a DNA sequence with increased Fis binding affinity. We used two sequences with different binding affinities (a high-affinity

sequence with K_d = 0.2 nM and a low-affinity sequence with K_d = 140 nM) from Chiu *et al.*, 2017 as an example. We took the lower affinity sequence as the input to DNAdesign, chose MGW as the shape parameter, and selected shape focal points from nucleotide positions -4 to +4. From the output of DNAdesign, we identified the top three candidates that minimize the central MGW. We found that all three candidates harbor the mutation from a C/G rich center to A/T rich at positions -2 and +2. One candidate matches the higher affinity sequence with the AATTT center (**Supplementary Fig. 2–3**).

S3.2 Design shape-perturbing DNA oligos to test for DNA shape preference of GTGCAC-binding TF AP2

The Apicomplexan Apetala 2 (ApiAP2) family is the largest and best-characterized TF family in the human malaria parasite *Plasmodium falciparum*. AP2, the DNA-binding domain of ApiAP2, can bind to either CACACA or GTGCAC DNA sequence motifs. Bonnell *et al.* found that the GTGCAC-binding AP2 binds to three extended motifs (AGAGCATTA, GGTGCAC, and TGTGCAC) with distinct DNA shape readout patterns. To test if shape readout changes lead to altered AP2-DNA binding, Bonnell *et al.* selected DNA oligos to maximize shape readout change while controlling the number of point mutations (Bonnell *et al.*, 2024). The authors performed EMSA with designed DNA oligos and concluded that AP2 binding to a high-affinity site does require specific shapes of the distal flanking sequence.

DNAdesign is perfectly suited to assist researchers with such design needs. Based on Bonnell *et al.*, we input GAGACCAGTGCATTATTAGTT as the wild-type sequence to DNAdesign, selected the six positions flanking the AGTGCATTA core as mutation sites, and choose MGW as the DNA shape parameter. We chose Levenshtein distance as the metric for base distance as in Bonnell *et al*. From the output of DNAdesign, we show that the mutation designs used in Bonnell *et al.* are indeed among the top shape mutation candidates given a required number of point mutations (**Supplementary Fig. 4**). We want to highlight that DNAdesign also provides a global comparison of all 4^7-1 possible mutation designs for this application, allowing a straightforward and comprehensive view for selecting desired mutation oligos.

Supplementary References

Bachmann,M. (2021) python-Levenshtein: Python extension for computing string edit distances and similarities. https://pypi.org/project/python-Levenshtein/

Bonnell,V.A., Zhang,Y., Brown,A.S. *et al.* (2024) DNA sequence and chromatin differentiate sequence-specific transcription factor binding in the human malaria parasite *Plasmodium falciparum*. *Nucleic Acids Res.*, **52**, 10161–10179.

Chiu,T.P., Rao,S., Mann,R.S. *et al.* (2017) Genome-wide prediction of minor-groove electrostatic potential enables biophysical modeling of protein–DNA binding. *Nucleic Acids Res.*, **45**, 12565–12576.

Chiu,T.P., Rao,S., and Rohs,R. (2023) Physicochemical models of protein–DNA binding with standard and modified base pairs. *Proc. Natl. Acad. Sci. USA*, **120**, e2205796120.

Hancock,S.P., Ghane,T., Cascio,D. *et al.* (2013) Control of DNA minor groove width and Fis protein binding by the purine 2-amino group. *Nucleic Acids Res.*, **41**, 6750–6760.

Hancock,S.P., Stella,S., Cascio,D. *et al.* (2016) DNA sequence determinants controlling affinity, stability and shape of DNA complexes bound by the nucleoid protein Fis. *PLoS One*, **11**, e0150189.

Zhou,T., Yang,L., Lu,Y. *et al.* (2013) DNAshape: a method for the high-throughput prediction of DNA structural features on a genomic scale. *Nucleic Acids Res.*, **41**, W56–W62.

Supplementary Figures

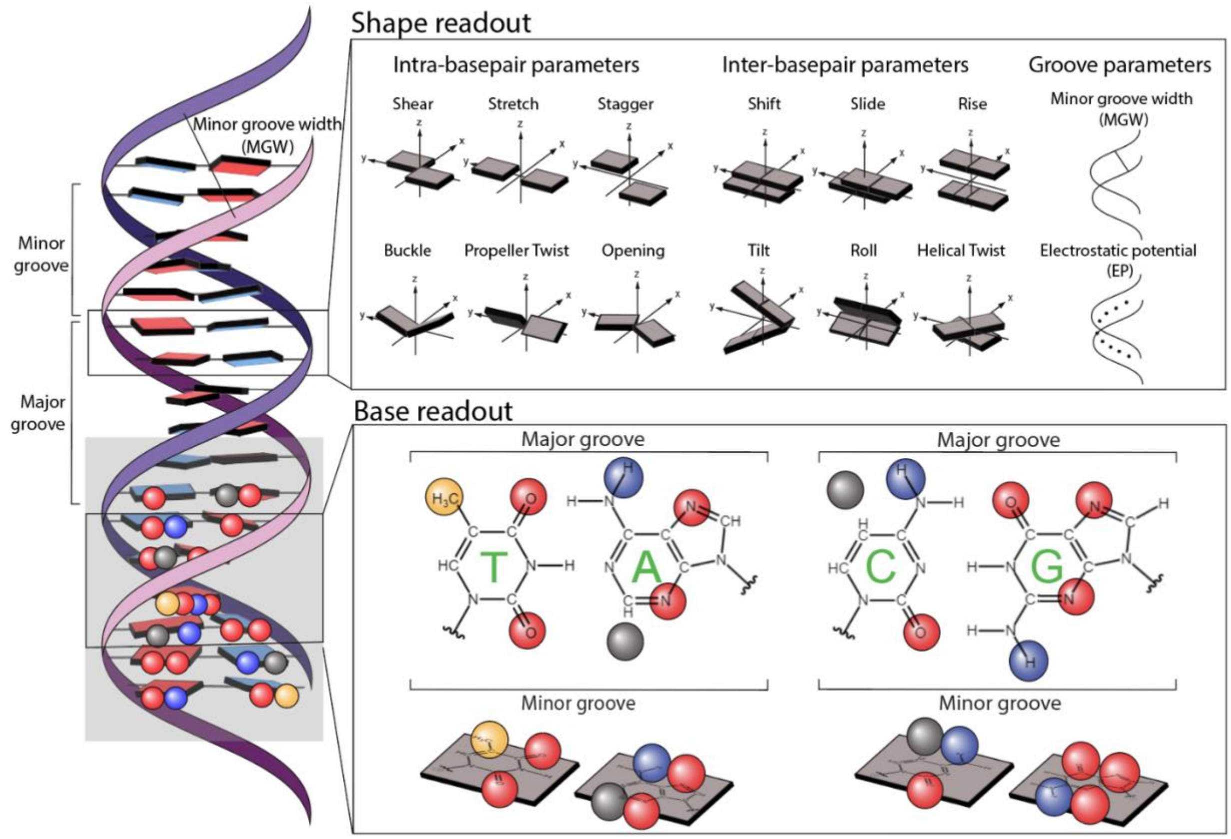

Supplementary Fig. 1: DNA shape readout and base readout. DNA shape refers to a set of 14 metrics (six intra-base pair parameters, six inter-base pair parameters and two minor groove parameters) that describe the structural and biophysical measurements of double-helical DNA. For DNA base readout, each base pair is represented as the functional groups at the base-pair edges that form direct contacts with amino acids. The functional groups include hydrogen bond donor, hydrogen bond acceptor, non-polar hydrogen, and methyl group.

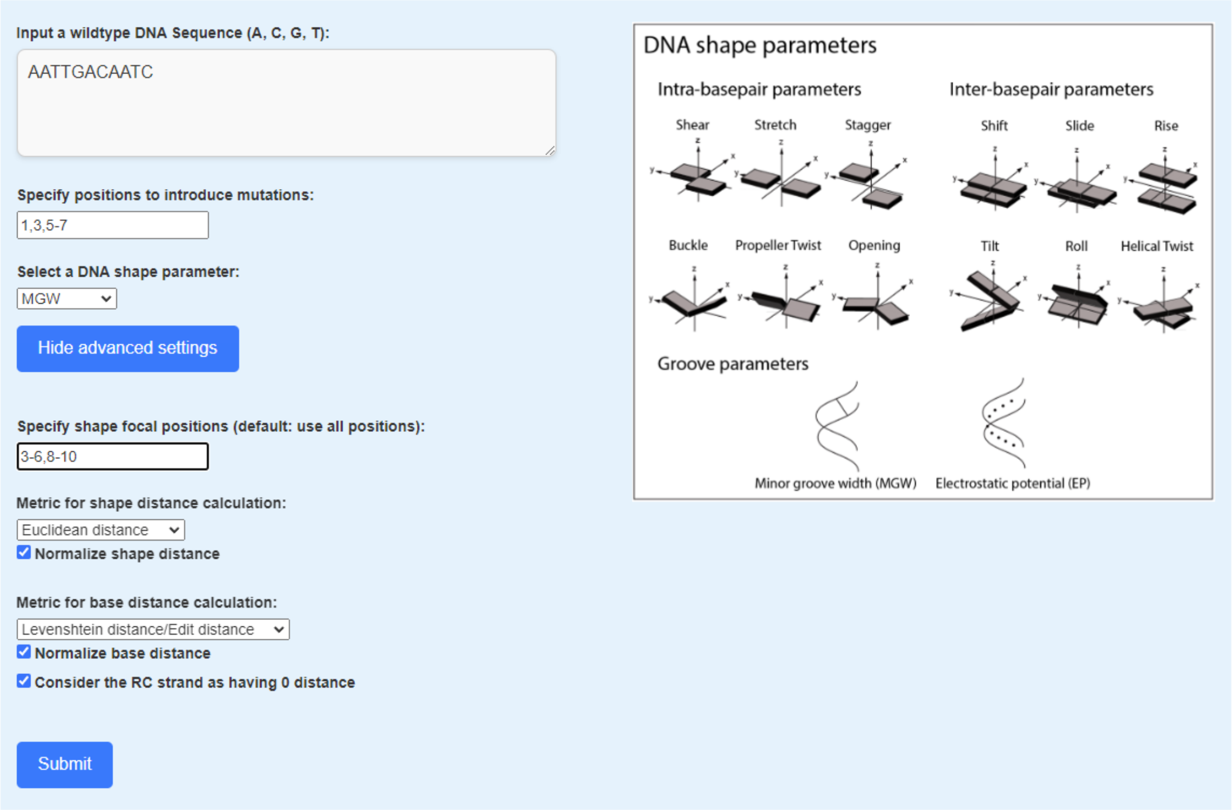

Supplementary Fig. 2: DNAdesign user interface and customizable advanced settings.

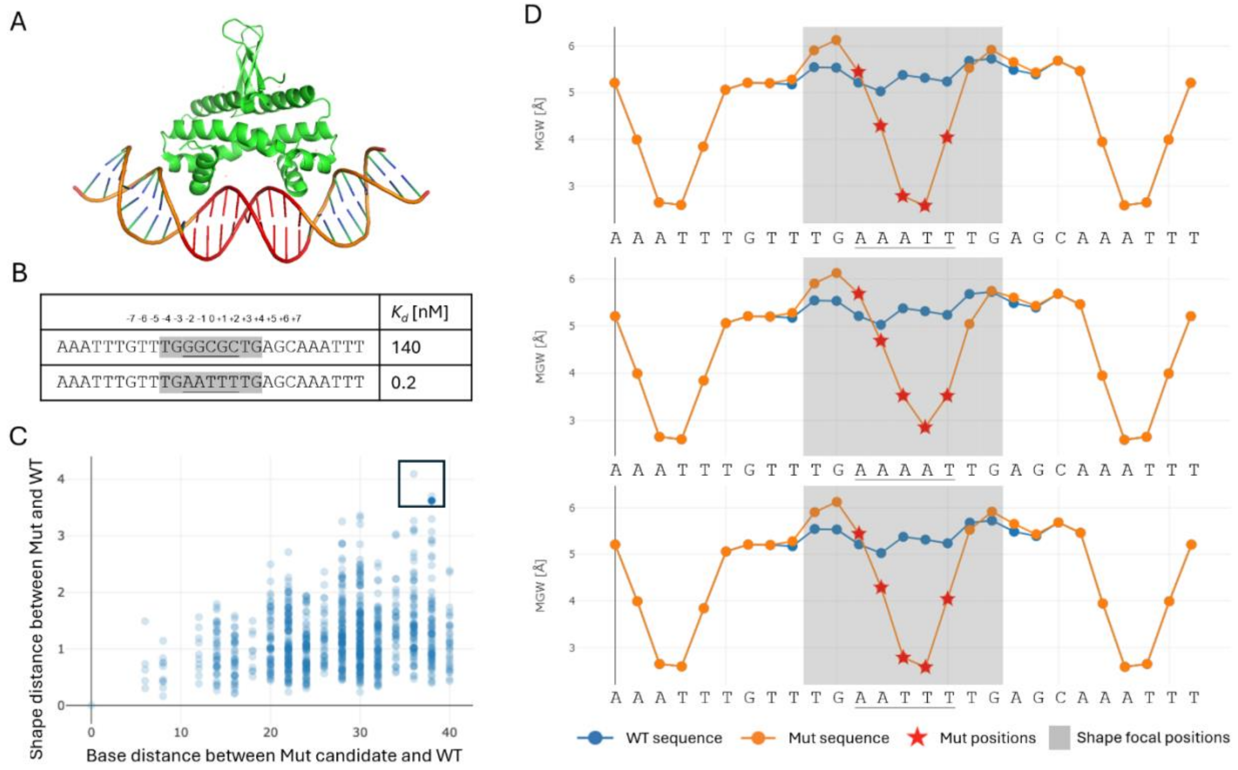

Supplementary Fig. 3: Application of DNAdesign for designing a high-affinity Fis binding site. (A) Co-crystal structure of a Fis–DNA complex (PDB ID: 3IV5), shape focal points shown in red. (B) Low and high affinity Fis-binding DNA sequences with shape focal points shaded in grey. (C) Base readout distance and shape readout distance plot output generated with DNAdesign, three mutation candidates that maximize the shape distance to wild-type are circled in the black box. (D) DNA shape readout plots comparing the MGW profile of each mutation candidate to the low-affinity sequence.

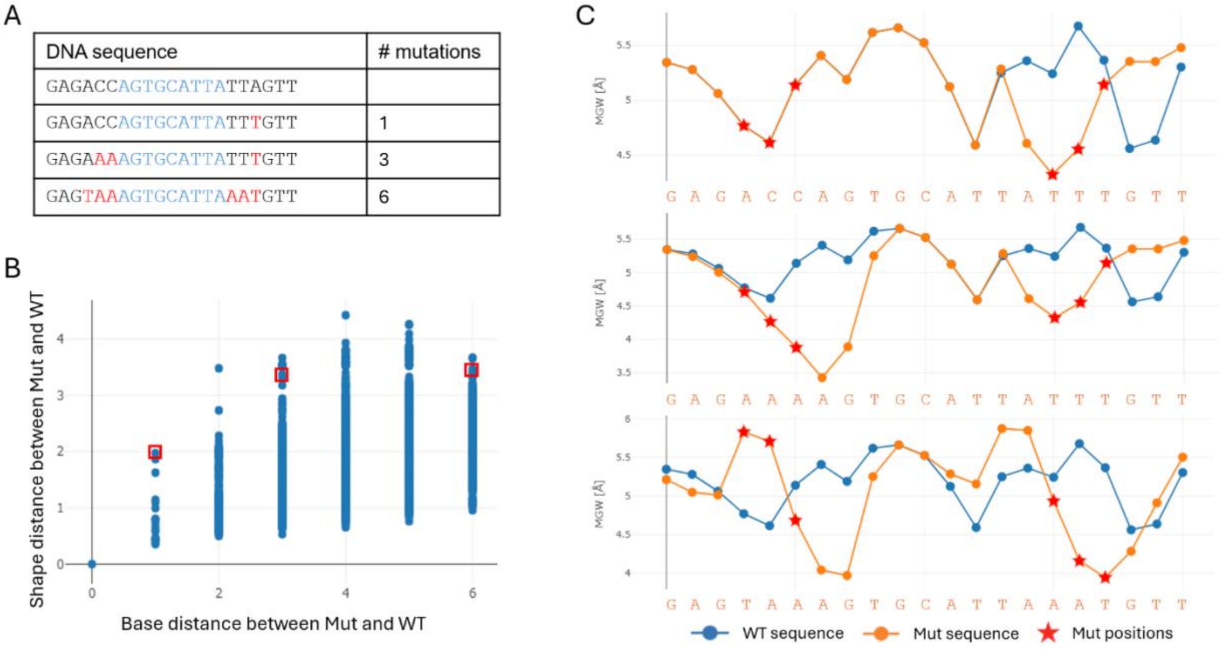

Supplementary Fig. 4: Application of DNAdesign for designing shape-perturbing DNA oligos to test for DNA shape preference of GTGCAC-binding TF AP2. (A) DNA oligos used by Bonnell *et al*. in their experiment. (B) Base readout distance and shape readout distance plot from DNAdesign using Levenshtein distance as the base-pair distance metric. Oligos in (A) are marked in red boxes. (C) DNA shape readout plots comparing the MGW profile of each mutation candidate to the wild-type sequence.
